# Adapting an Evidence-Based HIV Prevention Intervention Targeting High-Risk Migrant Workers: The Process and Outcome of Formative Research

**DOI:** 10.3389/fpubh.2016.00061

**Published:** 2016-03-31

**Authors:** Roman Shrestha, Pramila Karki, Santosh Pandey, Michael Copenhaver

**Affiliations:** ^1^Department of Community Medicine and Health Care, University of Connecticut Health Center, Farmington, CT, USA; ^2^Institute for Collaboration on Health, Intervention, and Policy, Storrs, CT, USA; ^3^HIV Prevention Project, Aasaman Nepal, Lalitpur, Nepal; ^4^Department of Allied Health Sciences, University of Connecticut, Farmington, CT, USA

**Keywords:** migrant workers, HIV prevention, HIV risk behaviors, evidence-based intervention, Nepal

## Abstract

**Background:**

Historically, HIV prevention efforts in Nepal have primarily focused on heterosexual transmission, particularly, among female sex workers and their male clients, with little acknowledgment of the contribution of migrant workers to the epidemic. The very few HIV prevention efforts that have been attempted with migrants have been unsuccessful primarily due to stigma, discrimination, and insufficient availability of culturally relevant evidence-based interventions (EBIs). As an initial step toward addressing this unmet need, we conducted formative research aimed at adapting an evidence-based HIV risk-reduction intervention for implementation among migrants in Nepal.

**Methods:**

Our formative work involved a critical examination of established EBIs and associated published reports complemented by data elicited through structured interviews with members of the target population and key stakeholders. Between July and August, 2014, we conducted structured one-on-one interview with migrants (*n* = 5) and key stakeholder (e.g., counselors, field workers, and project coordinator; *n* = 5), which focused on the HIV risk profiles of the migrants and on ways to optimize intervention content, delivery, and placement within the community-based settings. Data analysis followed a thematic analysis approach utilizing several qualitative data analysis techniques, including inductive analysis, cross-case analysis, and analytical coding of textual data.

**Results:**

Based on formative research, we adapted the Holistic Health Recovery Program, an EBI, to consist of four 30-min sessions that cover a range of topics relevant to migrants in Nepal. The intervention was adapted with flexibility, so that it could be provided in an individual format, implemented within or outside the community-based organization, and it can be delivered in either consecutive or weekly sessions based on time constraints.

**Conclusion:**

This paper provides a detailed description of the formative research process in preparation for the adaptation of an EBI – taking into account both empirical evidence and input from target population and key stakeholders – for use with migrants in Nepal. We hope that this study will help to inform similar work in the future as a growing number of EBIs have become widely available, but may not yet be in optimal form for implementation in real-world community-based settings.

## Introduction

The migration of people across national borders for employment purposes is a growing phenomenon and an increasingly important aspect of global, regional, and national economies (1). In the past decade, international labor migration has been the subject of wide ranging academic literature, and a growing body of evidence has shown it to be one of the social factors fueling the global HIV epidemic. Several studies have documented that migrants have a higher rate of sexual risk behaviors (2–8) and drug use behaviors (2, 3, 7–9), leading to their increased vulnerability to HIV infection. HIV transmission from high-risk populations to migrant workers and, in turn, to their native sexual partners is accelerating globally. It is widely believed that migrants tend to acquire infection at destination countries because of their engagement in risky behaviors. Upon return to their home country, they continue to have sexual contact with their partners, thus serving as a bridge population for spreading HIV from destination areas to their place of origin (3, 10).

Nepal, with a population of just over 26 million, has not been spared from the HIV pandemic (11). Nepal, where labor migration plays an important part in the national economy (12), has a concentrated HIV epidemic (13). The National Center for AIDS and STD Control (NCASC) reported that labor migrants were the principal driver of nation’s HIV epidemic, with 27% of the total estimated HIV infections attributed to this group (13). As in other parts of the world, studies in Nepal have documented that migrants have a higher rate of sexual risk behaviors and drug use behaviors leading to their increased susceptibility to HIV (2, 3, 14–17). The linkage between migration and HIV transmission risk behaviors explains the highly concentrated HIV epidemic among migrants in Nepal, and it has clear implications for HIV prevention, so that HIV is not widely transmitted to other risk populations.

In addition, our preliminary study conducted, in collaboration with a local NGO in Nepal, among returnee migrant workers indicated that migrants had significantly lower levels of education and were more-at-risk as they had less knowledge about HIV/AIDS, were more likely to engage in sex with female sex workers (FSWs), had higher number of sex-partners, had early coital-debut (before age 20), and frequently consumed alcohol or drugs before sex (18). The findings from this study support the need for HIV prevention interventions in regions with high male out-migration, with the aim of increasing HIV-related knowledge, awareness of personal vulnerability to HIV infection, and reduction of risk behaviors. These interventions would need to be tailored to focus on the populations most at risk for HIV – potential, active, and returnee migrants and their sexual partners.

Historically, HIV prevention efforts in Nepal have primarily focused on heterosexual transmission, particularly, among FSWs and their male clients, with little acknowledgment of the complex contribution of migrants to the epidemic. The very few HIV prevention efforts that have been attempted with migrants have been unsuccessful primarily due to stigma, discrimination, and insufficient availability of culturally relevant evidence-based interventions (EBIs) (19). Furthermore, most of the HIV prevention interventions that are available are often resource demanding or are otherwise incompatible for implementation among migrant communities. Fortunately, behavioral interventions have a long history of demonstrating that they are efficacious and adaptable tools for various types of health behavior change. Thus, driven both by contextual factors (e.g., resource limitations) and participant factors (e.g., inability or unwillingness to participate in lengthy and complex interventions), we aimed to adapt and refine the evidence-based HIV prevention intervention for use among Nepali migrant workers. In order to develop a culturally sensitive intervention, we incorporated Wiley’s framework – that includes accommodation, incorporation, and adaptation (20) – as the three initial courses of action for working with the migrant workers. As an initial step toward addressing this unmet need, we conducted formative research aimed at adapting an evidence-based HIV risk-reduction intervention for implementation among migrants. The complete process and our findings, including the adapted intervention, are outlined below.

## Materials and Methods

### Formative Research: Review of EBIs

In preparation for the adaptation of an evidence-based HIV risk-reduction intervention targeting migrants in Nepal, we conducted a formative research that first involved reviewing the available EBIs^1^ and associated published reports. Our goal was to select an EBI that was most relevant to migrants, the majority of whom have sexual- and drug use-risk behaviors. We also aimed to select an EBI that could be easily adapted as necessary for implementation among migrants within community settings in Nepal.

#### Selecting an EBI

As in prior studies (21, 22), we systematically reviewed all the EBIs (see text footnote 1) that were pertinent to the target population and rank ordered each of them based on the extent to which an intervention: (1) included content intended to address sexual- and drug use-risk behaviors, (2) is theory based, (3) has been applied to a range of population, and (4) is adaptable, if required, or can be applied in the original form. Using this assessment approach, we concluded that the Holistic Health Recovery Program (HHRP) (23) would be an ideal fit for HIV prevention intervention targeting high-risk migrants in Nepal.

The HHRP is a psychotherapeutic intervention dedicated to harm reduction, health promotion, and improved quality of life (QOL) for individuals addicted to illicit drugs. It is comprised of 12 2-h weekly manual-guided group sessions with comprehensive HIV risk-reduction content that addresses the medical, emotional, and spiritual needs of drug-involved individuals (see Table 1). Each session is designed to last 2 h and is cofacilitated by two trained facilitators. Cofacilitators address individuals’ potential motivational conflicts by providing them with self-protective as well as altruistic reasons for examining and changing their HIV risk behavior.^2^ The HHRP is based on the information–motivation–behavioral (IMB) skills model (24), which has demonstrated to be effective in improving behavioral outcomes – including reduced drug- and sex-related risk behaviors – when tested in randomized controlled trials (RCTs) in community-based settings (23). Moreover, a feasibility study within a community-based treatment setting indicated that HHRP intervention could be considerably condensed and adapted to accommodate real-world organizational constraints without undue loss of intervention potency (21).

**Table 1:** **Outline of the Holistic Health Recovery Program (HHRP) intervention**.

No.	Group topic	Skills taught
1.	Reaching your goals	Improving memory and concentration, setting goals, establishing priorities, action initiation
2.	Health-care participation	Understanding immune system, HIV, hepatitis B, and hepatitis C, improving skills for establishing and maintaining a partnership with health-care providers, strategies for improving adherence to medical recommendations
3.	Reducing the harm of injection drug use	Identifying the harm of injection drug use, learning harm reduction techniques, reducing cue-elicited craving
4.	Sexual harm reduction with latex	Identifying the harm of unsafe sexual practices, learning harm reduction techniques
5.	Negotiating harm reduction with partners	Harm reduction negotiation and communication skills
6.	Preventing relapse to risky behavior	Creating a road map for the journey of recovery, learning relapse prevention skills, identifying early warning signs, understanding seemingly irrelevant decisions
7.	Healthy lifestyle choices	Coping skills, stress management, nutritional guidelines, and food hygiene
8.	Introduction to the 12 steps	Identifying what is and is not controllable, understanding when to let go and when to take action, identifying one’s personal source of strength, increasing motivation for change
9.	Overcoming stigma	Understanding the consequence of stigmatization, decreasing the strength of “addict” self-identity, connecting with “core/ideal” self, redefining the self as a non-drug user
10.	Motivation: overcoming helplessness	Understanding the source and consequences of helplessness, identifying situations in which you can become empowered, assessing readiness for change, increasing motivation to pursue a healthy lifestyle
11.	Emotional and spiritual healing	Understanding the stages of grief, understanding and managing anger and depression, facing and coping with fear, finding personal meaning
12.	Healthy social relationships and activities	Identifying and maintaining healthy social relationships, identifying and engaging in health social activities

#### Adapting an EBI

As our aim was to implement the HRRP intervention in a different setting, it was essential to take cultural context (e.g., diverse values, beliefs, and behaviors) into account, in tailoring intervention to the migrants. In order for our intervention to be culturally competent, we incorporated Wiley’s framework, which includes accommodation, incorporation, and adaptation as the three initial courses of action for working with the migrants (20). *Accommodation* required us to have a better understanding of the communicative styles and literacy practices of migrants and to account for such factors during intervention adaptation. Because our research team included native Nepalese with experience conducting community-based projects with the migrants in Nepal, we were readily able to engage in this course of action throughout the intervention development process. *Incorporation* required us to become familiar with community practices and to incorporate them in the intervention. *Adaptation* involved approaching the intervention development process with the philosophy of conveying tailored information and skills to reduce risk behaviors among migrants (20).

### Formative Research: Elicitation Interviews

We employed the Assessment-Decision-Administration-Production-Topical experts-Integration-Training-Testing (ADAPT-ITT) framework (25) as a guide for systematically adapting HHRP intervention. We collected information from members of the target population (i.e., migrants) and key stakeholders (i.e., counselors, field workers, and project coordinator) (see Tables 2 and 3). The objective of conducting elicitation interviews was to guide the adaptation process, in terms of determining (1) intervention content (i.e., specific content areas of the original HHRP modules to include/exclude, emphasize/abbreviate), (2) delivery modality (i.e., group vs. individual), and (3) duration (i.e., length/timing).

**Table 2:** **Structured interview instrument for collecting data from the target population participants**.

Item	Question
1.	What are the key health issues you had while you were working abroad? Has that changed since you returned to Nepal?
2.	Have you received HIV-related information from any source (i.e., school, NGO, mass media)? If so, was it helpful?
3.	Can you tell me what you know about HIV?
4.	Tell me about the ways HIV can be transmitted?
5.	Tell me how one can know if he/she has HIV?
6.	Tell me how one can prevent getting or spreading HIV?
7.	Do you know your HIV status? Do you know where one can get tested for HIV?
8.	Do you think that migrant workers engage in risky behaviors such as unsafe sexual and drug use behaviors? If so, are they safe?
9.	Were you sexually active while working abroad? What about since you returned to Nepal? Why?
10.	Do you like using condoms? What do you like/not like about condoms?
11.	Have you consumed illegal drug?
12.	Do you inject drugs? If so, do you share needles or do you clean needles or get new needles?
13.	Do you think that programs for HIV education are helpful? What would be the most helpful?
14.	What type of intervention would work best: individual or group (Aasaman Nepal’s office) or individual (home)?
15.	How long should each session last?
16.	Do you think it would be better to have your family member involved in the intervention? Why?

**Table 3:** **Structured interview instrument for collecting data from the key stakeholders**.

Item	Question
1.	Do you think your patients have sufficient information on HIV, safe sex, and injecting practices?
2.	What types of HIV risk behaviors (sex and drug related) do you perceive in your patients?
3.	What attitudes toward safer sexual and drug behaviors do your patients possess?
4.	What norms do patients have that interfere with safer sexual and drug using behaviors?
5.	Do you think there may be any deficits in behavioral skills that may contribute to any risky behavior?
6.	Do you counsel your patients on HIV risk reduction? If so, what are the challenges you have experienced?
7.	What approaches do you use that may be helping to increase patients’ HIV preventive behaviors?
8.	How do you feel about the educational program on HIV for your patients?
9.	What type of intervention would work best (e.g., individual, group)?
10.	How long should each session last?
11.	What materials can or cannot be used for the purpose of the intervention?

#### Participants

Between July 12 and August 10, 2014, we conducted structured one-on-one interview with migrants (*n* = 5) and key stakeholders (*n* = 5). Migrants were volunteers who were confidentially recruited from the local community-based organization (CBO) based on the degree to which they matched our target population of “returnee migrant worker.” Returnee migrant worker is defined as “*an individual who had been to a foreign country for employment purpose and has returned to Nepal at least three months prior to the study*.” Inclusion criteria of our target population are (1) returnee migrant workers, (2) 18 years or older, and (3) report a history of HIV risk behaviors (i.e., sex- and/or drug-related risk behaviors) within past 6 months. Key stakeholders included counselors, field workers, and project coordinator within the CBO in Ramechhap district, Nepal, where migrants seek counseling and treatment. Key stakeholders were recruited based on the degree to which they assisted in the HIV prevention program. The demographic characteristics of the target population and key stakeholders are presented in Table 4.

**Table 4:** **Demographic characteristics of all interview participants**.

	Target population (*n* **=** 5)	Key stakeholders (*n* **=** 5)
Gender	Male (4)	Male (3)
Age	21–34 years (mean: 26 years)	23–38 years (mean: 29 years)
Educational status	Primary (1)	High-school (2)
	Secondary (2)	Bachelors (2)
	High-school (1)	Masters (1)
	Bachelors (1)	
Countries visited	Malaysia (2)	n/a
	Saudi Arabia (1)	
	Qatar (1)	
	UAE (1)	
Occupation	Domestic service (1)	Coordinator (1)
	Driver (1)	Counselor (2)
	Construction (2)	Field worker (2)
	Cook (1)	
Work experience	3–8 years (mean: 5.2 years)	1–6 years (mean: 4.4 years)

#### Instrument and Procedures

As in prior work (22), we used brief, structured but relatively open-ended instruments to collect data (Tables 2 and 3). All participants were informed that the objective was to elicit a range of information that could collectively guide the refinement of an HIV risk-reduction program that could be optimally implemented among migrants. Thus, some items focused on the HIV risk profiles of the migrants, whereas other items focused on ways to optimize intervention content, delivery, and placement within the community-based settings. All interviews were audio-tape recorded and transcribed in Nepali and translated to English. The English translation was back-translated to Nepali to ensure appropriate translation. Trained doctoral level researcher conducted interviews under the supervision of a licensed clinical psychologist. The study protocol was approved by the Investigational Review Board (IRB) at the University of Connecticut and received board approval from Aasaman Nepal. After determination of eligibility, the investigator provided an overview of the protocol and obtained consent from each participant on one-to-one basis. Since the study included educationally disadvantaged participants, the consent was obtained verbally in native (Nepali) language from all participants.

#### Analytic Approach

We used Atlas.ti software to facilitate management and analysis of data (26). Data analysis followed a thematic analysis approach utilizing several qualitative data analysis techniques, including inductive analysis, cross-case analysis, and analytical coding of textual data. Initial inductive analyses involved discovering emergent themes and patterns within the dataset to develop a preliminary project codebook. From this preliminary codebook, code names and definition evolved to match emerging data during iterative analyses of the interviews by research team members. Two research team members (Roman Shrestha and Pramila Karki) met regularly to build coding consensus, to become familiar with participant narratives, to contextualize discrepancies, and to make coding and cross-case analysis decisions of newly uncovered themes. The primary themes identified formed the basis of the selection of an appropriate intervention and the subsequent adaptation of the intervention to meet the specific needs of the target population. The identified primary themes are summarized in the section below.

## Results

### Interviews with the Target Population

#### Primary Health Issues Faced While Abroad

The majority of participants reported that they suffered from some types of health problem while working abroad. The most common types of health issues they faced were “*headache*,” *fever*,” “*gastrointestinal illness*,” “*respiratory symptoms*,” and “*accidental injuries*.” Typical responses from the participants included

Usually, I suffered from headache … I had one minor accident. Luckily, nothing major happened … I sometimes had difficulty breathing. It used to be too hot, much hotter than here [Nepal]. One of my friends died. They said he died due to heart attack.

When asked whether they still had those health issues since returning to Nepal, responses included

Not anymore. But every now and then I get it [headache] … Sometimes, yes [stomach problem] … No, I haven’t had any accident here in Nepal.

#### Knowledge about HIV

When asked about HIV, all respondents reported to have heard about HIV. They received information primarily through an “*educational institution*,” “*mass media* (e.g., *radio*, *television*, and *print media*),” and “*outreach programs conducted by CBO*.” Most participants reported to have heard before going abroad, whereas one respondent reported

I heard it [HIV] through SaMi people [counselor from Safer Migration project]. Before I went abroad [Qatar], I had no idea. I came back to Nepal. I came here [Ramechhap district] to get a new passport, then people from the SaMi told us about it [HIV].

When asked about what they knew about HIV, the majority of migrants responded HIV as “*an incurable disease*,” “*a sexually transmitted infection*,” and “*fighting power of body goes away*.” Typical responses were

People say it [HIV] has no cure, right? … I heard in a radio that it [HIV] is a STD … This disease [HIV] takes away the fighting power of human body. That’s why, infected person has to take medicine all their life.

Furthermore, HIV was perceived as “*the end of life*” or “*death sentence*” by some participants. The association of HIV with the death may be due to the fact that HIV is perceived as an “*incurable disease*,” and people think very often that infection with HIV means the “end of life.”

My doctor said there is no cure to it [HIV]. It’s an end of life. Death sentence!

#### Ways of HIV Transmission

“*Unprotected sexual intercourse*” was mentioned as the main way of HIV transmission. Additionally, the respondents also mentioned “*blood transfusion*,” “*having sex with sex worker (FSW)*,” “*using drugs*,” and “*mother to her child*” as the ways of HIV transmission. Several participants were unaware that HIV transmission can occur through injection drug use. There was also some misinformation of how HIV was transmitted. Some participants indicated that they believed HIV could be transmitted *via* “*saliva*,” “*sharing utensils and clothes*,” “*mosquito-bite*,” and “*kissing*.”

One can get it [HIV] from getting bad [HIV-infected] blood, having relationship with FSWs, saliva, and I think also from mosquito-bite … Yes, through blood, while having sex, kissing.

In the discussion about common ways to prevent getting or spreading HIV, all participants knew it was important to “*always use condom*,” though only few reported to have always used condom in the last month. None of the participants mentioned about using clean or new needle to shoot drugs but on participant reported

I’ve never used Ganja, Chares, or Heroin [drugs]. I don’t know how, but, people say it [using drugs] can transmit HIV.

Similarly, only one participant mentioned about the “*vertical transmission*” of HIV from infected mother to her unborn child.

… also transmission from mother-to-child. Therefore, HIV infected women should not be pregnant.

#### HIV Testing

When asked about their HIV status, only one participant mentioned having tested for HIV. Almost all of the participants knew that they could get tested for HIV in “*hospital*”; however, they chose not to because of stigma, fear, or ignorance. Typical responses included

No, never tested for HIV. This hospital in Chautara [district hospital] does it [HIV testing] but it’s no use to me. I haven’t had sex with FSWs … No, because I don’t want people to look at me suspiciously … Yes, one time before I left for abroad [Malaysia]. It was negative …

#### HIV Risk Behaviors

When questioned about common types of HIV risk behaviors migrants engage in while abroad, the majority of the participants reported that they believed that many of the migrants engage in “*sexual relationship with FSWs*” with “*minimal condom use*.” When prompted if they had “*sex after drinking alcohol*,” the majority of participants responded affirmatively. Common responses included

I never went to brothel. Many others know it [having sex with FSWs] is bad, but, they still do it. And they do not use condom … I had sex with FSW a couple of times. I think, I used condom both the times … We get lonely there [abroad]. After working so hard, we need to enjoy our life too, right? … I had some female friends there [India] with whom I had sex. I never felt like using condom then.

When questioned about reasons to not using condoms, participants stated “*price*,” “*feeling uncomfortable to buy*,” “*difficulty negotiating condom use with their partner*,” and “*decrease in sexual pleasure*” as the primary barriers.

I don’t like using it [condom]. I can’t feel the same pleasure and it [condom] is expensive too … I don’t feel comfortable to go to the shop to buy condom. It’s embarrassing, you know?

With regard to illegal drug use behavior, none of the participants reported to have used any illicit drug, but one participant reported that he had one friend who used illicit drug while abroad.

Never in my life … No, it’s illegal there. If police finds out that [using illicit drug] you’ll be sent to jail for a long time … My friend did [use drug], but I never did. He offered me once but I told him, no.

#### Intervention Content and Delivery

We asked participants for ideas to help design an appropriate HIV risk-reduction intervention that would be most helpful for implementation among migrants. First, we sought information about whether they thought that an HIV prevention intervention would be feasible and helpful for them. All the participants responded quite positively.

Absolutely … Yes. Many people still don’t know much about HIV … Yes, this will really help people to be informed about HIV and to stay healthy.

Thus, participants tended to strongly endorse the concept of including an HIV prevention intervention to be specifically tailored for migrants.

When asked to provide any other information, we should consider in attempting to create an optimal HIV prevention intervention, most participants suggested that they would prefer participating in an individual sessions at home rather than a group-level intervention in Aasaman Nepal’s facility because of concerns about “*discussing sensitive topics*” and “*lack of transportation*.” Typical responses were

I feel I cannot open up while talking about HIV and other things in front of other people. Ramechhap bazaar [location of Aasaman Nepal’s office] is far from my house … I don’t mind both, but like one-to-one more. I will feel comfortable to ask questions. Place-wise, I like getting it [intervention] close to my house when there is no one around …

Participants also offered their views about how the intervention content should be delivered. The participants liked the idea of using videos and PowerPoint presentations as teaching tools but most of them suggested the use of “*color printed handouts*” in a “*simple to understand language*” would be more feasible. This was due to lack of required infrastructure and resources (e.g., adequate space, laptops, projector, and screen) in the place where the intervention is to be delivered.

I like videos but I don’t think that will always be possible here. We have load-shedding [power outage] everyday … Handouts will be easier for you to do, I think. Because, where are you going to do your work [presentation] Will you be able to carry everything with you?

When asked about how long each session should last, the majority of migrants suggested that they would prefer intervention to be “*brief*,” as exemplified by

I prefer short sessions. Because we’ve so many other things to do at home … Short sessions, between 30 minutes to an hour, will be better.

Participants also expressed that their family members should have knowledge about fundamentals of HIV. However, when asked about whether or not family member should be involved in intervention, the participants preferred not to.

It is extremely important for my family to know about it [HIV]. But I feel uncomfortable to discuss about it [HIV] with them by my side.

### Interviews with the Key Stakeholders

#### Their Patients’ Knowledge about HIV and Risk Reduction

The key stakeholders indicated that their patients have relatively “*lower level of information*” about HIV and risk reduction.

Most of the migrant workers don’t have sufficient information about HIV. They do not know all about how it is transmitted and can be prevented … People usually have the information about the importance of condom use. They know these things but they have wrong information too. For example, many still believe that saliva or mosquito-bite transmit HIV.

They all agreed that the migrants tend to practice relatively higher level of HIV risk behaviors. However, they did report that they are more concerned about their sex-risk behavior, as indicated by

The migrants are using condom less often … They [migrants] engage in paid sex with FSWs while abroad. I’ve noticed that drug use among them is low.

#### Their Patients’ Attitudes about Risk-Reduction Behaviors

When questioned about migrants’ attitudes regarding risk-reduction behaviors, some responses were

Many of them [migrants] know about the risk of engaging in unsafe sex but they still do. They think condom use is unnecessary … Some feel it [condom use] is not satisfying …. Some worry about being untrustworthy.

This implies that the key stakeholders felt that a key barrier to engage in condom use for migrant workers is that some think it is “*unnecessary*,” some felt that sex would be “*less pleasurable*” due to condom use, and some may imply “*unfaithfulness*” to their spouse or primary partner.

Regarding drug-related behavior, all stakeholders tended to agree that drug-related behaviors were not an issue with migrants and that they were less concerned about it. Some of their comments were

No, I don’t see they [migrants] use drugs. Some of them have this problem but it is very low … Probably, they [migrants] are scared of getting into legal problems, that’s why very few use drugs.

#### Intervention Content and Delivery Style

The key stakeholders tended to agree that most migrants have condom application skills; however, they typically lack the skills to negotiate safer sex with their partner. Some stakeholders also mentioned that the migrants do not feel comfortable buying condoms, especially when they encounter someone of the opposite sex in the shop.

They [migrants] have difficulty asking their partner to use a condom. Partner may find it offensive … One time my patient mentioned that he doesn’t buy condoms because he feels very shy to ask the shopkeeper for condoms, particularly when shopkeeper is a female.

Four of the stakeholders, who were counselors and field workers, reported that they routinely talk to their patients about HIV, how HIV is transmitted, and what measures can be implemented to prevent from getting HIV.

Yes, I give them [migrants] information. I do a weekly field trip and that’s when I talk to them … Every time I get a new person [migrant] to counsel, I talk to him/her about HIV/AIDS.

When asked about the challenges they have encountered during counseling, they mentioned “*lack of time*,” “*privacy*,” and “*confidentiality*” as the key factors.

They [migrants] say they don’t have time for this [to attend program]. Some find it hard to share sensitive information with others.

Aside from these issues, few stakeholders also mentioned that “*uneasiness*” to share sexual behavior information to opposite sex and presence of “*stigma*” could be barriers, as captured by

Most of the time, females feel uncomfortable to discuss about HIV and other sensitive information with me [male counselor] … Seeing counselor or participating in a program does not look good. Other people start thinking that something must be wrong with that person.

To overcome these barriers, they usually meet with the migrants in a “*private room*” for a “*short-time*” and convince them that “*confidentiality*” will be maintained. Also, they offer gender-matched counseling.

The stakeholders highlighted the importance of HIV education program, especially to this vulnerable population.

It [HIV educational program] is very important. You need to focus on migrants … We just have one HIV program here [Ramechhap district], I think we need more. It’s very hard for one project to cover all areas.

Overall, the stakeholders indicated that “*one-on-one sessions*” would better fit with our target population, taking into account the circumstances and resources available. They also stressed “*gender matching*” to foster rapport and comfort between patients and counselors.

Personally, I’d like to conduct group sessions. But that will be too hard to do. You’ll have to go to their houses to educate them [migrants]. So, individual sessions will better fit the circumstances. Also, it’ll be best to gender match counselor/field trip worker to the patient.

In addition, they suggested that the intervention should be relatively “*brief*” and carefully “*tailored to accommodate participants’ knowledge*, *educational level*, and *risk behavior experiences*.” In relation to this, the stakeholders suggested to “*provide handouts*” and “*show colorful pictures*” related to the intervention content to the participants.

Make sure intervention is brief and to the point. Too long is bad. They won’t have time for that. Doing presentations, showing videos would be really informative and appealing to the patients. But I don’t think they will come here [Aasaman Nepal’s facility] for the intervention. We’ll have to go to their house. And there, all these are not possible. So, providing handouts and verbally educating them is the best option. Colorful handouts in an easily understandable language is even better.

### Intervention Adaptation

The findings of this formative research among returnee migrant workers and key stakeholders (e.g., counselors, field workers, and project coordinator) indicated that it would be most suitable to implement an adapted version of the evidence-based HHRP (23) for our target population of migrant workers in Nepal, rather than implementing it or any other existing EBI in the original form. Given the parameters, it was more appropriate to shorten the original HHRP (23) content to emphasize the fundamentals of HIV, HIV risk reduction, certain interpersonal risk-reduction skills (e.g., negotiating risk reduction with partners) and to restructure the delivery techniques, so that the intervention could be conducted in individual sessions. In terms of intervention content, suggestions indicated that greater emphasis should also be placed on the HIV risk profiles, particularly related to migrants. Importantly, we sought to preserve the process of the original HHRP (23), while integrating only the essential changes as suggested by the formative data.

Additionally, we looked at issues concerning the strategic placement of the intervention. This process was mostly dictated by resource constraints, logistics of the collaborating CBO, and feasibility among participants, as described in the interviews. The key aspects that we considered were (1) how this EBI could be made available to the largest group of migrants, (2) how it could be positioned to be perceived by migrants and key stakeholders as relevant to migrants’ general well-being, (3) how it could be least disruptive to the migrants’ activities of daily living and to the CBOs routine, and (4) how it could be employed so that it would be most likely to be sustained using available resources within the target area and CBOs.

The resulting intervention, Holistic Health Recovery Program for Nepal (HHRP-N), consists of four one-on-one 30-min sessions intended to cover a range of applicable topics (Table 5) addressing the HIV risk behavior faced by Nepalese migrants who go abroad for employment purposes. Importantly, it is designed with flexibility, so that it could be provided in an individual format, implemented within or outside the CBO, and it can be delivered in either consecutive or weekly sessions based on time constraints. Regardless of intervention placement, the content will emphasize HIV prevention as a crucial part of migrants’ healthy living.

**Table 5:** **Outline of the HIV risk reduction intervention that resulted from formative research with target population and key stakeholder participants**.

No.	Group topic	Skills taught
1.	Active health-care participation	Understanding HIV, strategies for improving health, testing and linkage to care, establishing and maintaining a partnership with health-care providers, enhancing adherence to medical recommendations
2.	Reducing HIV-related risk	Identifying sex- and drug-related HIV-risks, learning about proper harm reduction techniques
3.	Negotiating risk reduction with partner	Establishing trust, negotiating use of condom, communication about sex- and drug-related HIV risk
4.	Overcoming stigma	Understanding the consequence of stigmatization, overcoming stigma, and discrimination

## Discussion

This study contributes to a growing body of literature describing strategies and process to address critical issues that may arise during intervention adaptation and implementation (22, 25, 27, 28). It provides a detailed description of the formative research process in preparation for the adaptation of an EBI for use with migrants in Nepal. The resulting HHRP-N is a behavioral intervention that has been designed to address the HIV risk behavior faced by Nepalese migrants who go abroad for employment purposes.

The results of this formative study suggest that there is a great need for HIV risk-reduction interventions, primarily focused on sex-related risk reduction, tailored for migrant workers in Nepali context. Also, it is evident that there is extensive room for improvement in migrants’ level of knowledge about HIV and risk reduction, and in their motivation and skills to engage in safer sexual practices. For example, the report of inconsistent condom use during sexual intercourse with FSWs indicates a significant need for HIV prevention among this population.

Study participants identified several barriers (e.g., resource constraints, logistics of the collaborating CBO, accessibility to participants) to the feasibility and sustainability of the intervention that we were able to address in the adaptation process. We employed the Wiley’s global framework – that includes accommodation, incorporation, and adaptation – to develop culturally sensitive intervention (20). In addition, we applied the ADAPT-ITT model (25) to guide the adaptation process, in which input from the migrants and key stakeholders (e.g., counselors, field workers, and project coordinator) was incorporated into the intervention adaptation. The evidence gathered from this formative research had a significant impact on the features of the resulting HHRP-N intervention, including the intervention content, delivery modality, and length. As suggested by prior research (21), this information was critical in enabling us to refine an intervention perceived to be applicable to migrants as well as practical in terms of situational circumstances and local organizational demands.

The common challenge that researchers usually face during the intervention adaptation phase is preserving the overall efficacy of the adapted intervention among the target population while simultaneously accommodating the resource constraints of real-world settings, as we have described. It is likely that various organizations or research groups may utilize the same EBI and a similar adaptation approach; however, the overall outcome of the process would be expected to differ as a function of the target population, local resource constraints, and organizational demands, as suggested by this study.

Although our study provides insight into the adaptation of a widely available EBI, some of the study limitations should be noted in order to place our findings in the proper context. Foremost, this study was conducted with the objective of adapting an EBI in a community-based setting, as opposed to conducting a study with quantitative research outcomes, such as a RCT, and this resulted in the limitations that are inherent in qualitative research. However, we believe that our selection of participants, a well-established analytical approach, and the incorporation of published empirical research, resulted in well-informed decisions in the intervention adaptation process. Second, the data provide a rich context to the overall intervention adaptation process although the small sample size may limit our ability to generalize the findings to a different risk population. Third, the adapted version of the HHRP intervention has never been tested in the Nepali context, and it is not clear whether it will have any positive effects on migrants in Nepal. However, similar studies have already been successfully deployed in prison and community-based treatment settings and have shown to be feasible, acceptable, and effective in reducing HIV risk behaviors (29, 30). Fourth, the gender perspective on migrants is an important issue, as male and female migrant workers may face different opportunities and vulnerabilities during their migration. However, gender-disaggregated data were not collected, which may have limited our ability to address gender-specific needs and expectations in the adapted intervention. Finally, this formative research did not collect data on social network and perceived and available social support of the migrant workers was not collected, which has a significant impact on their health while abroad. Regardless of the noted limitations, this study points to the potential benefits of systematically adapting EBI across international setting by taking cultural context into account, as well as other issues unique to a particular setting.

## Conclusion

This paper provides a detailed description of the formative research process in preparation for the adaptation of an EBI – taking into account both empirical evidence and input from target population and key stakeholders – for use with migrants in Nepal. The findings of this formative study suggest that there is a great need for HIV risk-reduction interventions, primarily focused on sex-related risk reduction, tailored for migrant workers in Nepali context. The resulting HHRP-N is a behavioral intervention designed to address the HIV-related risk behaviors faced by Nepalese migrants who go abroad for employment purposes. There is a clear need for research and translating research into practice to ensure that EBIs are successfully implemented where they are needed most, even if this means adapting an original EBI in order to enhance intervention feasibility and acceptability. We hope that the process and outcome of this formative research will help to inform similar work in the future as a growing number of EBIs have become widely available (see text footnote 1), but may not yet be in optimal form for implementation in real-world community-based settings.

## Author Contributions

RS and MC contributed substantially to the conception and design of the study. All authors contributed to the analysis and interpretation of the data, and critical review and revision of the manuscript. They have given final approval of the version to be published and agreed to be accountable for all aspects of the work. RS and MC also acquired funding and coordinated the study.

## Conflict of Interest Statement

The authors declare that the research was conducted in the absence of any commercial or financial relationships that could be construed as a potential conflict of interest.

## Footnotes

^1^https://effectiveinterventions.cdc.gov/

^2^http://medicine.yale.edu/spiritualselfschema/training/hhrp/hhrpgroup.aspx
